# Rivaroxaban Effects Illustrate the Underestimated Importance of Activated Platelets in Thrombin Generation Assessed by Calibrated Automated Thrombography

**DOI:** 10.3390/jcm8111990

**Published:** 2019-11-15

**Authors:** Stephanie Makhoul, Marina Panova-Noeva, Véronique Regnault, Wolfram Ruf, Philip Wenzel, Jeremy Lagrange

**Affiliations:** 1 Center for Thrombosis and Hemostasis, University Medical Center Mainz, 55131 Mainz, Germany; stephaniemakhoul@live.com (S.M.); Marina.Panova-Noeva@unimedizin-mainz.de (M.P.-N.); ruf@uni-mainz.de (W.R.); wenzelp@uni-mainz.de (P.W.); 2 DZHK (German Center for Cadiovascular Research), Partner Site Rhine Main, University Medical Center Mainz, 55131 Mainz, Germany; 3 Université de Lorraine, INSERM U1116, DCAC, 54505 Nancy, France; veronique.regnault@inserm.fr; 4 CHRU Nancy, Vandœuvre-lès-Nancy, 54511 Nancy, France; 5 Department of Immunology and Microbial Science, The Scripps Research institute, La Jolla, CA 92037, USA; 6 Center for Cardiology—Cardiology I, University Medical Center Mainz, 55131 Mainz, Germany

**Keywords:** thrombin, platelets, factor Xa inhibitor, phospholipids

## Abstract

Background: The direct oral anticoagulant rivaroxaban inhibiting specifically activated factor X (FXa) causes delayed thrombin generation (TG) as measured by calibrated automated thrombography (CAT). The implications of these changes for assessing bleeding or residual prothrombotic risks of patients are unclear in the absence of a better understanding of the underlying mechanism. Methods: We compared platelet rich plasma (PRP) without or with prior collagen-induced platelet aggregation (agPRP) in the CAT assay to better characterize TG in the presence of rivaroxaban. Results: In the presence of rivaroxaban, TG curves in agPRP showed a distinct profile with a rapidly ascending phase followed with a protracted phase. Inhibition of tissue factor pathway inhibitor amplified the first phase of the curve which was also modulated by procoagulant phospholipids. Inhibition of FXIIa-dependent FXI activation revealed that aggregated platelets influenced the first phase by a combination of extrinsic and intrinsic coagulation pathway initiations. Thrombin-dependent amplification of TG (even prior collagen activation) was responsible for the second phase of the TG curve. Conclusions: AgPRP fully includes platelet ability to support TG and reveal distinct TG phases in the presence of direct FXa inhibitors highlighting its potential use in an anticoagulated setting.

## 1. Introduction

Direct oral anticoagulants (DOACs) are increasingly used as first line treatment for venous thromboembolism (VTE) and for prevention of ischemic stroke in atrial fibrillation, but regular assessment of their anticoagulant effects remains challenging. The coagulation system leads to the formation of thrombin and fibrin and its polymerization that will form a clot. Most of the thrombin generation (TG) occurs after the clot formation and total TG potential of a plasma can be assessed with calibrated automated thrombography (CAT), which gives a better overview of the coagulation system than clotting times (prothrombin time or activated partial thromboplastin time) [1,2,3,4]. The CAT assay uses a fluorogenic substrate to measure prothrombin conversion to thrombin in a recalcified plasma in the presence of phospholipids (PL). In patients with acute VTE, the use of TG assays becomes challenging once an antithrombotic treatment, including DOACs, has been initiated. It is well established that anticoagulants impair dramatically thrombin generation, making it difficult to monitor the bleeding or residual prothrombotic risk.

The COMPASS study (Cardiovascular Outcomes for Peoples Using Anticoagulant Strategies) included patients with stable atherosclerotic disease treated with rivaroxaban plus aspirin compared to rivaroxaban or aspirin alone and showed a lower risk of cardiovascular death, stroke, myocardial infarction, fatal bleeding, or symptomatic bleeding into a critical organ with dual therapy [5]. However, major bleeding events occurred in 3.1% (rivaroxaban plus aspirin patients) vs. 1.9% (patients under aspirin) pointing out that even if the combination of anticoagulation with antiplatelet therapy on the overall cardiovascular risk is beneficial, stratification needs to be improved.

Current clinical guidelines recommend assessment of bleeding and thrombotic risks when making a decision to start anticoagulation, principally by using the clinical risk scores, such as ABC-bleeding [6], HAS-BLED [7], or CHA2DS2-VASc risk score [8]. In addition to clinical characteristics, biomarkers shown to improve bleeding and/or stroke risk estimates are also part of the clinical scores, such as NT-proBNP, high-sensitivity cardiac troponin T (hsTnT), growth differentiation factor-15 (GDF-15), hemoglobin level and platelet count [6,9]. However, risk scores may present limitations depending on cohort recruitment and follow-up and parameters selected [10]. In addition, to date no hemostatic functional assays are routinely used to estimate patients’ bleeding risk. The evaluation of bleeding risk in patients receiving both anticoagulant and antiplatelet agents, e.g., rivaroxaban and aspirin, poses a greater challenge. The reason is that the hemostasis inhibition includes both inhibition of platelets and of coagulation, and most laboratory tests evaluate these processes separately.

The presence of rivaroxaban in plasma can change the appearance of the TG curve with the formation of camel-back shaped instead of normal curves [11]. Endogenous thrombin potential (ETP), representing the total amount of thrombin formed, and the peak height of TG, representing the maximum thrombin formed, are the most widely used parameters to evaluate the thrombin potential of a plasma. However, the presence of DOACs seems to affect more the parameters related to the kinetics of TG such as the lag time, the velocity and the time to peak, often not reported in the literature [12]. Not only the amount (ETP) but also the kinetic of thrombin formation and its inhibition are important for hemostasis. After a systemic review using the words (thrombin generation) and (rivaroxaban) in PubMed, from the 54 publications released over the past 10 years in which the CAT assay was performed only 26 presented curves of TG and no more than 17 of these publications showed camel-back shaped curves. These curves were also described with other Xa inhibitors as apixaban [13]; however, to date this effect has not been clarified. To the best of our knowledge, no specific studies reported on the impact of rivaroxaban on the shape of TG curves.

In the present work, we aimed to explore the origin and determinants of camel-back shaped TG curves in the presence of new oral anticoagulants, in particular rivaroxaban. We took advantage of the use of aggregated platelet rich plasma (PRP/agPRP) in the CAT [14] which fully includes platelet procoagulant activity since modified TG kinetics in particular faster TG could compensate the slow TG often observed after rivaroxaban treatment.

## 2. Experimental Section

### 2.1. Blood Sampling and PRP Preparation

Studies using human platelets were approved by the ethics committee of the University Mainz (study no. 837.302.12; 25.07.12). Blood from 16 healthy volunteers (8 women, 8 men, mean age 30 ± 2.6 years) was obtained after informed consent by venipuncture via a 21-gauge needle and collected in S-monovette tubes containing sodium citrate (3.2%) (Sarstedt, Nümbrecht, Germany). Complete blood counts and hematocrit were determined with a cell counter (KX-21N, Sysmex Corporation, Kobe, Japan). PRP was prepared within 1 h following blood sampling with a centrifugation at 200× *g* for 10 min at 20 °C. The supernatant was removed and the platelet count was adjusted to 200 × 109/L with platelet poor plasma (PPP) prepared with a 10 min centrifugation at 2000× *g* at 20 °C. Platelet free plasma (PFP) was prepared by centrifuging PPP at 13,000× *g* for 30 min at 4 °C. The collected PFP was stored at −80 °C until use.

### 2.2. Platelet Aggregation

Platelet aggregation was monitored for 10 min by measuring light transmission through stirred PRP at 37 °C using a platelet aggregometer (APACT 4S plus aggregometer, Diasys Greiner, Holzheim, Germany). Platelet aggregation was triggered by 2 µg/mL equine collagen fibrils type I (Chrono-log Corp, Havertown, PA, USA). Aggregated PRP was used directly after aggregation for CAT measurement [14].

### 2.3. Thrombin Generation Assay

CAT was performed with PRP and agPRP or PFP (Figure S1). CAT in PRP and PFP was performed in a microtiter plate fluorometer (Fluoroskan Ascent, ThermoLabsystems, Helsinki, Finland) using dedicated software (Thrombinoscope BV, Maastricht, The Netherlands) as reported previously [15,16]. All reagents were used as follows: 80 µL PRP, homogenous agPRP or PFP, 20 µL of PRP-Reagent or PPP-Reagent low (Thrombinoscope BV, Maastricht, The Netherlands), and 20 µL of a mix of fluorogenic substrate and calcium (FluCa-Kit reagent). In specific experiments with PRP or agPRP TG was triggered with 0.1 U/mL human thrombin (Roche Diagnostics, Mannheim, Germany) instead of PRP-Reagent. Rivaroxaban, PAR 1 (vorapaxar) and PAR 4 (BMS 986120) inhibitors were purchased from Cayman Chemical (Ann Arbor, MI, USA) and reconstituted in DMSO. Vorapaxar was used at a final concentration of 100 ng/mL following a 10,000-times dilution in HEPES buffer (HEPES 20 mM, NaCl 140 mM, pH 7.35) before an additional 67 times dilution in plasma. BMS 986120 was used at a final concentration of 1 µg/mL following a 1000 times dilution in HEPES buffer before an additional 67 times dilution in plasma. Anti-TFPI antibody was purchased from Loxo (Dossenheim, Germany) and used at a final concentration of 5 µg/mL. An IgG isotype control was purchased from Sigma-Aldrich, St Louis, MO, USA) and used at a final concentration of 5 µg/mL (Figure S2). The 14E11 monoclonal antibody which is directed against the A2 domain of FXI in order to block its activation by FXIIa was provided by A. Gruber and use at a final concentration of 5 µg/mL. The phospholipid vesicles (PV) consisted of phosphatidyl-choline -serine -ethanolamine (PC/PS/PE; Sigma-Aldrich, St Louis, MO, USA) 60/20/20 mol % at final concentrations of respectively 1 µM, 4 µM, and 48 µM equivalent PS were prepared in HEPES buffer sonicated (amplitude 8%) 5 times 5 min on ice bath.

### 2.4. Statistical Analysis

Normal distribution of the data was tested by D’Agnostino and Pearson test. Data were analyzed with Kruskal–Wallis or Mann–Whitney test. Results are presented as median (min–max).

## 3. Results

### 3.1. Addition of Rivaroxaban to agPRP Change the Shape of the TG Curve

**Figure 1:**
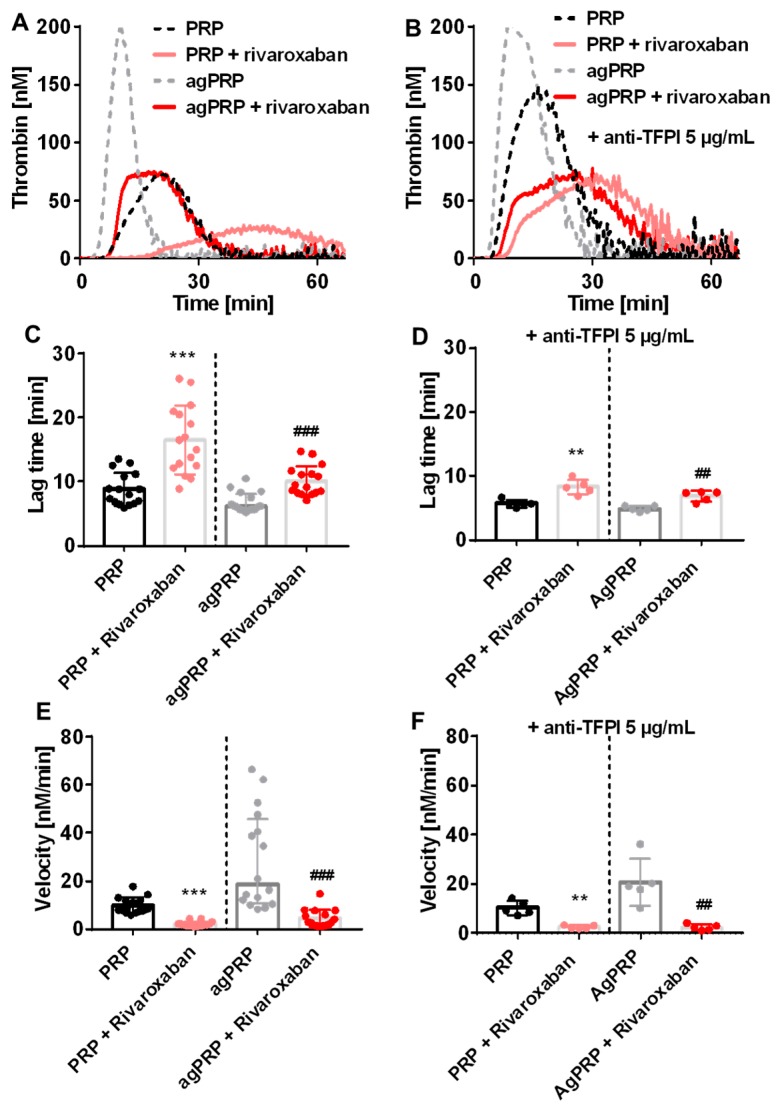
Effect of platelet aggregation with addition of rivaroxaban and anti-tissue factor pathway inhibitor (TFPI) on human platelet rich plasma (PRP) in thrombin generation (TG). Representative TG curves in PRP or PRP after aggregation (agPRP) triggered with 2 µg/mL of collagen with and without addition or 50 ng/mL of rivaroxaban (**A**) and inhibition of TFPI (anti-TFPI 5 µg/mL) (**B**). Lag time of thrombin generation for the different conditions (**C**,**D**). Velocity of thrombin generation under the different conditions (**E**,**F**). Results are presented as median (min-max). ** *p* < 0.01, *** *p* < 0.001, vs. PRP; ## *p* < 0.01, ### *p* < 0.001, vs. agPRP, *n* = 16 per group for left panels and *n* = 5 for right panels.

TG was performed in PRP and collagen-induced agPRP from healthy volunteers in the absence or presence of rivaroxaban and initiated with 1 pM of tissue factor (TF) (Figure 1A). Addition of rivaroxaban to PRP leads to two-fold increased lag time (8.4 (5.9–13) versus 16 (8.9–26) min) and time to peak (tt peak) (18 (14–25) versus (35 (28–51) min) and decreased peak and velocity of TG (Table 1, Figure 1C,E). ETP is also significantly decreased (Table 1). The use of agPRP spiked with rivaroxaban showed similar modifications (Table 1, Figure 1D,F). Concerning the curve shapes, PRP and agPRP presented normal shaped TG curves (Figure 1A). After addition of rivaroxaban to PRP, small curve alterations were discernible in a few subjects while clear transformation of the TG curve into a camelback shape was visible when using agPRP.

### 3.2. TFPI Modulates the First Phase of the Camelback TG Curves

TG curves in agPRP in the presence of rivaroxaban and initiated with 1 pM TF presented a camelback shape with two peaks. One possible explanation is the ability of TFPI to inhibit both TF-FVIIa and FXa. In order to limit these effects an antibody targeting TFPI was added prior the CAT assay (Figure 1B). In the presence of rivaroxaban, inhibition of TFPI affected lag time and velocity to the same extent as without rivaroxaban but camelback curves were still present (Figure 1D,F). Variation of ETP, peak and tt peak with or without rivaroxaban after collagen-induced agPRP were similar as the variations observed without TFPI inhibition (Table 1). Interestingly, TFPI inhibition exacerbated the first phase of the camelback phase in PRP plus rivaroxaban leading to curves similar to agPRP plus rivaroxaban.

### 3.3. Phospholipids Modulate the Camelback Shape of TG Curve in Presence of Rivaroxaban

**Figure 2:**
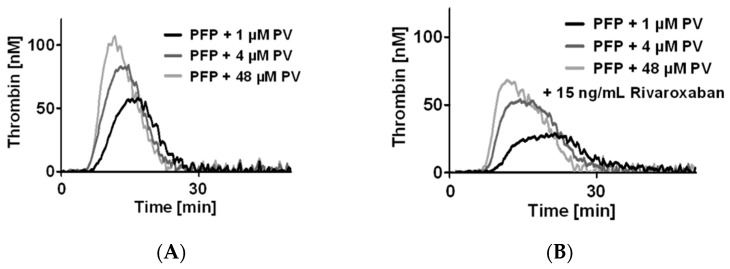

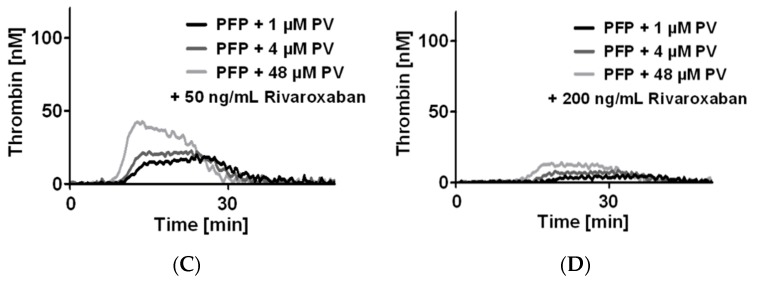
Modulation of camel-back shaped thrombin generation (TG) curve by the addition of phospholipids artificial vesicles. Representative curves of TG in platelet free plasma (PFP) supplemented with increasing concentrations of rivaroxaban: baseline (**A**), 15 ng/mL (**B**), 50 ng/mL (**C**), and 200 ng/mL (**D**). For each concentration, three concentrations of phospholipid vesicles (PV) were tested. PRP: Platelet rich plasma; agPRP with collagen 2 µg/mL, ETP, endogenous thrombin potential, tt peak, time to peak. Results presented as median (min-max). *n* = 6.

AgPRP can influence TG by the release of coagulation factors such as FV and emission of procoagulant vesicles. It seems unlikely that platelet-derived coagulation factors are implicated since camelback curves are often present when using PFP. To test whether PL could modulate the appearance of camelback curves we used PFP in which we added different concentrations of PV and rising concentrations of rivaroxaban (Figure 2). The addition of PV to PFP mimicked the camelback curves of TG especially when using the usual PV concentration used for the CAT assay (4 µM). PV increased the peak height of the first TG phase of the camelback shaped curve in a concentration-dependent manner. Moreover, a shift in the peak was visible, changing the peak from long and vague at low PV concentration to high and marked at high PV concentration (Figure 2B,C). This effect was visible in the changes of the modifications of the tt peak (Table 2).

### 3.4. A Potential Platelet Secondary Activation Is Not Responsible for the Camelback TG Curves

**Figure 3:**
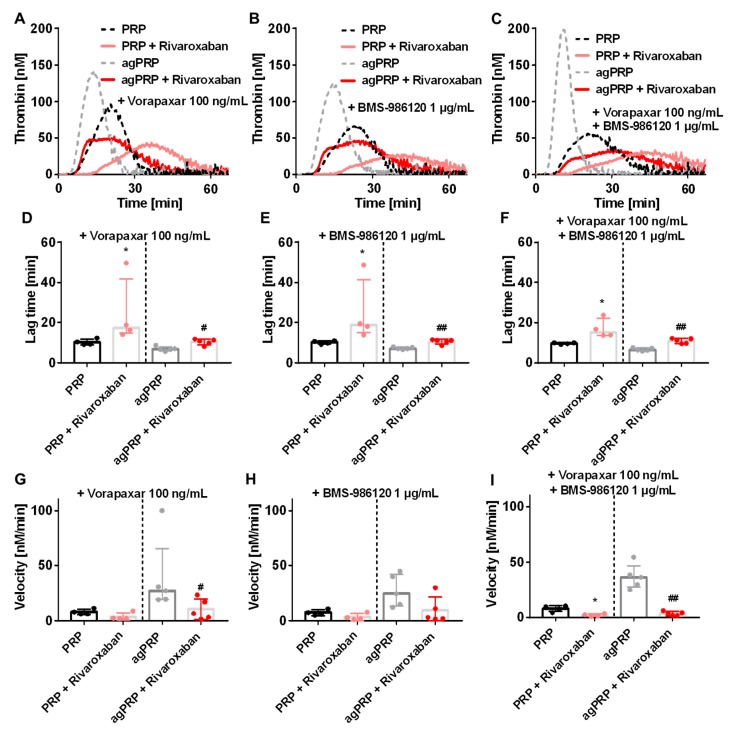
Effect of platelet aggregation with addition of rivaroxaban, PAR-1 and PAR-4 inhibitor to human platelet rich plasma (PRP) in TG. Representative TG curves in PRP or PRP after aggregation (agPRP) triggered with 2 µg/mL of collagen with and without addition or 50 ng/mL of rivaroxaban and inhibition of PAR-1 with vorapaxar (100 ng/mL) (**A**), inhibition of PAR-4 with BMS 896120 (1 µg/mL) (**B**) or a combination of both inhibitor (**C**). Lag time after addition of vorapaxar (**D**) BMS 986120 (**E**) or a combination of both inhibitor (**F**). Velocity after addition of vorapaxar (**G**) BMS 986120 (**H**) or a combination of both inhibitor (**I**). Results are presented as median (min-max). * *p* < 0.05, vs. PRP; # *p* < 0.05, ## *p* < 0.01, vs. agPRP, *n* = 4–5 per group.

Another possible explanation of the camelback curves is a secondary activation of platelets by the first amount of thrombin generated during the initiation of TG. In order to inhibit the thrombin effect on platelets we added PAR-1 (vorapaxar) and PAR-4 (BMS-986120) inhibitors or the combination of both prior the CAT assay (Figure 3). In PRP addition of PAR-1 and/or PAR-4 inhibitor prior to CAT tended to lower TG while no changes were visible after aggregation (Table 1). However, inhibition of PAR-1 and/or PAR-4 in the presence of rivaroxaban affected the kinetic parameters (lag time and velocity) to the same extent as without rivaroxaban (Figure 3D–I) and camelback curves were still present (Figure 3A–C).

### 3.5. Triggering TG with Thrombin in the Presence of Rivaroxaban Abolished the Camelback Curve

**Figure 4:**
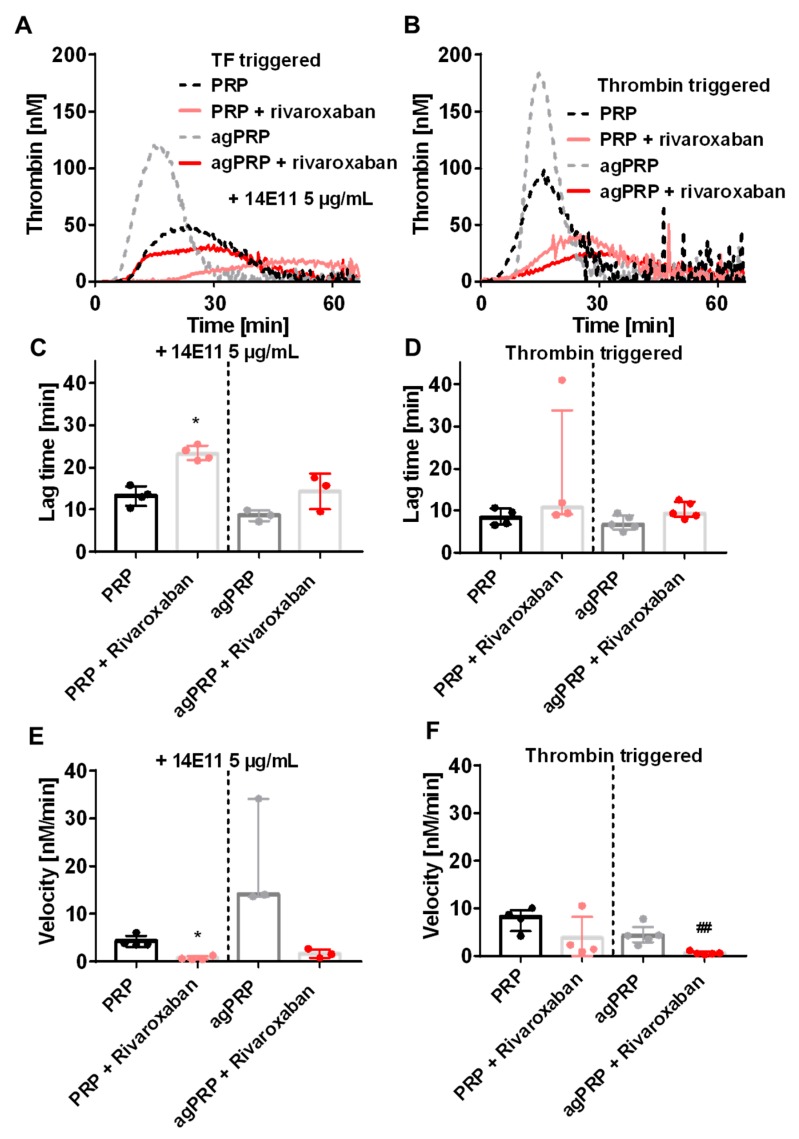
Implication of tissue factor (TF) and thrombin positive feedback pathways in the camel-back shaped TG curve. Representative TG curves in PRP or PRP after aggregation (agPRP) triggered with 2 µg/mL of collagen with an antibody inhibiting FXIIa-dependent activation of FXI without affecting thrombin-dependent activation of FXI (14E11; 5 µg/mL) (**A**). Corresponding lag time and velocity (**B**,**C**). Representative curves of PRP and agPRP triggered with thrombin instead of TF (**D**) and corresponding lag time and velocity (**E**,**F**). Results are presented as median (min-max). * *p* < 0.05, vs. PRP; ## *p* < 0.01, vs. agPRP. *n* = 3–5 per group.

The two phases of the rivaroxaban-derived TG curves could be the result of dissociation between TG amplification related to FVa and propagation phases due to activation of FXIa by thrombin. Indeed, FXa inhibition prolonged TG and could lead to a distinct appearance of those phases, which should normally be fused into the normal shaped TG curve. To test this, we used an antibody blocking specifically FXI activation by FXIIa but not by thrombin (antibody 14E11). In this condition, agPRP plus rivaroxaban still displayed camel-back curves with two phases in the ascending part of the curve (Figure 4A) but a lower first ascending phase compared to agPRP with rivaroxaban must be noted. Rivaroxaban displays similar effects on PRP and agPRP in the presence of 14E11 as in its absence (Table 1, Figure 4C,E). To highlight the thrombin-dependent increase of TG, we used low amounts of thrombin to initiate TG (Figure 4B). This lead to normal shaped curves in PRP as well as in agPRP with or without rivaroxaban despite a flattening of the curves indicating, in concert with the decrease of the first ascending part of TG in agPRP plus rivaroxaban with 14E11, that the first phase is a combination of the activation of both extrinsic and intrinsic pathways, while the latter phase depends on thrombin activation (Figure 4D).

## 4. Discussion

Recent studies reported an unexpected interaction between platelet and oral thrombin inhibitors leading to increased platelet aggregation during arterial thrombosis [17,18]. Platelets, especially mean platelet volume and platelet count, are important modulators of TG [19]. Interactions between platelets and the coagulation system are most often not considered in clinical laboratory tests and the use of direct oral anticoagulants presents novel challenge into this analytical gap. Here we explored the determinants of camelback TG curves appearing in the presence of FXa inhibitor. AgPRP was used as we observed that performing platelet aggregation before performing the CAT assay highly accelerates the kinetics of prothrombin conversion compared to PRP. Moreover, the observed changes were different compared to an activation step of platelet prior TG [14]. Thus, using PRP with unstimulated platelets does not fully include their procoagulant activity (induced by the first traces of generated thrombin) and ability to support thrombin generation. The use of agPRP in TG measurement is an advantage in the presence of anticoagulants that flatten TG curves and highly diminish ETP. Indeed, after addition of 50 ng/mL of rivaroxaban, TG curves were still visible when using agPRP and displayed two phases in the ascending part (or very long-lasting peaks). Some publications reported the presence of bell-shaped curves in patients taking rivaroxaban and we reproduced this phenomenon in control persons by adding ex vivo 50 ng/mL rivaroxaban [20,21,22]. The concentration of 50 ng/mL corresponds to the plasma concentration of rivaroxaban 12 h after administration of 10 mg of rivaroxaban [23]. Under physiological conditions, platelet activation mainly due to exposed collagen follows vascular injury and supports TG while in the CAT assay using PRP, platelets are activated once first traces of thrombin are generated. This can be visible by comparing lag time of TG which is often prolonged in PRP compared to similar conditions in PFP, while the opposite should be expected due to platelet procoagulant function and ability to generate procoagulant large extracellular vesicles and coagulation factors. In our set of experiments, baseline lag time for PRP was 8.9 ± 2.6 min vs. 7.5 ± 2.1 min (mean ± SD) for PFP. This shows that at baseline, platelets are neutral to coagulation and need to be activated to support thrombin generation.

In a comment, Kremers et al. [24] have suggested that TFPI could play a role in the appearance of the camel-back curves. We found that the addition of an antibody against TFPI did not restore normal shaped curves. However, in the presence of anti-TFPI the first phase of the ascending part of the curve in PRP in the presence of rivaroxaban matched the agPRP plus rivaroxaban curve indicating that contrary to the second phase of the camel-back shape the first is highly dependent on TF and FX. Since platelets contain almost half of circulating full length TFPI, which is the main active form, the absence of increased TG in agPRP plus rivaroxaban compared to PRP plus rivaroxaban in the presence of the anti-TFPI is surprising [25,26]. The increase in procoagulant PL may maximize the formation rate of thrombin and TFPI action is not sufficient to inhibit it.

In the presence of rivaroxaban, a secondary activation of platelets by the generated thrombin was a possible explanation for the occurrence of camelback curves once aggregation is completed. It has been already described that PAR-1 is responsible for a second wave of aggregation [27]. In order to test this hypothesis, we added inhibitors of platelet receptors for thrombin PAR-1 and PAR-4. The very first ascending phase of the curve was perceptibly decreased after the use of PAR-4 and in combination of PAR-1 inhibitors. However, these inhibitors—separately or combined—did not abrogated the camelback curves.

In order to test the importance of FXII and FXI in the first phase, we added an antibody (14E11) blocking specifically FXIIa-dependent FXI activation, or initiated TG directly with a small amount of thrombin. Addition of 14E11 did not abolish the first phase of camelback curves although the first phase peak height was lowered compared to identical conditions with no antibody. This result shows that both intrinsic (starting from thrombin-triggered FXI activation) and extrinsic pathways lead to this first burst of thrombin generation. Interestingly, the use of thrombin as a trigger of TG to dissect the importance of thrombin-dependent TG amplification in the presence camelback shape curve reveals that this amplification is not responsible for the presence of camel-back curves. Conversely to TF-triggered TG in PRP or agPRP, lag time and time to peak were identical when using thrombin as an initiating factor. These results highlight the importance of TF and FXIIa-dependent initiation in enhancing the kinetics of TG. Moreover, triggering PFP (where platelets are replaced by PV) with thrombin leads to no TG emphasizing the role of platelets in TG amplification which could be linked with recent findings pointing out the ability of platelet surface to concentrate procoagulant factors (FIXa, FX, and FXa, FVIII and prothrombin) [28]. This role of platelets is again not visible without prior activation. One remaining question was coming from the important variability observed in the first phase of the camelback shaped curves. Platelet-derived microvesicles are well described to initiate TG in a FXIIa-dependent manner [29]. Antiplatelet agents were shown to lead to two-peaked thrombin generation in PRP due to delayed PL exposure on the platelet surface [30]. Thus, we decided to add increasing concentrations of PV in PFP. The most striking effect of PV in the presence of rivaroxaban was on the first ascending phase of the curve indicating that surfaces, presenting PL such as platelet-derived microvesicles are very important for the first amplification of TG. Here again TG performed with PRP very likely hides the ability of platelets to modulate TG through microvesicle production.

Taken together, our results show that agPRP might be of use in the presence of new oral anticoagulant since TG is well detectable. The relevance of the CAT assay has been established in the occurrence of venous thrombosis, but its use is more controversial concerning the prediction of recurrence [31,32,33,34]. The use of agPRP could lead to a better stratification of thrombotic risk in patients by including platelet ability to modulate TG (independently of thrombin induced platelet activation) which is underestimated with the use of unactivated PRP. Taking into account the full procoagulant potential of platelets to TG in the CAT assay was already attempted more than 15 years ago by using frozen-thawed PRP and could be an alternative if agPRP cannot be prepared freshly [35]. Since the shape of the curve is affected, it will be necessary to reevaluate the current parameters of the CAT that were developed for asymmetric bell-shaped curves. Another aspect that deserves to be extensively studied is the intra and inter-individual variability to an identical dose of rivaroxaban which is very important, making the requirement for developing an integrative test to personalize treatment even more attractive [23,36,37].

## 5. Conclusions

In conclusion, we present evidence that camelback shaped curves in the presence of rivaroxaban result from a protraction of TG duration and dissociation of the amplification phase and the thrombin-dependent propagation phase. In addition, we show that the first phase can be modulated by PL and could potentially be of use for personalized treatment adjustment.

## Figures and Tables

**Table 1:** PRP thrombin generation parameters.

	**ETP (nM.min)**			
	**PRP**	**PRP + Rivaroxaban**	**agPRP**	**agPRP + Rivaroxaban**
TF	1489 (1231–2185)	1299 (819–1759) **	1680 (1437–2692)	1330 (1016–1915) ###
TF + anti-TFPI	1473 (1231–2185)	1375 (1175–1759)	1579 (1510–2692)	1324 (1016–1915)
TF + Vorapaxar	1348 (1196–1483)	1148 (546–1443)	1911 (1514–2130)	1461 (1220–1621) #
TF + BMS 986120	1332 (1190–1463)	977 (667–1423)	1737 (1481–2078)	1259 (1084–1753)
TF + Vorapaxar + BMS 986120	1414 (1231–1538)	1143 (779–1445)	1864 (1556–2095)	1363 (873–1519) ##
TF + 14E11	1124 (821–1370)	692 (570–1482)	1621 (1375–1727)	917 (719–1304)
Thrombin	1220 (873–1380)	794 (300–1002) *	834 (615–1086)	758 (610–1003)
	**Peak (nM)**			
	**PRP**	**PRP + Rivaroxaban**	**agPRP**	**agPRP + Rivaroxaban**
TF	91 (64–152)	35 (22–72) ***	137 (82–270)	48 (26–87) ###
TF + anti-TFPI	85 (64–115)	36 (34–49) **	105 (91–219)	36 (28–66) ##
TF + Vorapaxar	79 (64–106)	38 (24–46) *	182 (140–328)	52 (33–101) ##
TF + BMS 986120	75 (66–98)	28 (26–49) *	168 (110–227)	47 (34–129) #
TF + Vorapaxar + BMS 986120	88 (57–99)	35 (29–58)	199 (171–245)	37 (30–61) ##
TF + 14E11	49 (45–73)	16 (12–29) *	120 (104–183)	29 (15–42)
Thrombin	70 (43–92)	21 (15–38) *	33 (28–56)	13 (11–24) ##
	**tt peak (min)**			
	**PRP**	**PRP + Rivaroxaban**	**agPRP**	**agPRP + Rivaroxaban**
TF	18 (14–25)	35 (18–51) ***	14 (9.7–19)	26 (15–40) ###
TF + anti-TFPI	17 (16–18)	35 (30–37)	14 (12–16)	29 (22–31)
TF + Vorapaxar	21 (19–22)	39 (32–72) *	14 (9.0–15)	20 (14–34) #
TF + BMS 986120	22 (1–23)	39 (30–72) *	13 (12–17)	26 (15–33)
TF + Vorapaxar + BMS 986120	21 (19–24)	38 (29–72) *	12 (11–13)	25 (20–32) ##
TF + 14E11	26 (24–28)	49 (47–50) *	16 (15–16)	33 (28–36)
Thrombin	18 (14–21)	30 (26–58) *	15 (12–22)	35 (30–49) ##

Thrombin generation parameters of platelet rich plasma (PRP) and PRP after aggregation (agPRP) triggered with 1 pM of TF or thrombin (0.1 U/mL) with addition of rivaroxaban (50 ng/mL), anti-TFPI (5 µg/mL), vorapaxar (100 ng/mL), BMS 968120 (1 µg/mL), or 14E11 (5 µg/mL). Results are presented as median (min-max). *n* = 3–16, * *p* < 0.05 ** *p* < 0.01 *** *p* < 0.001, vs. PRP, # *p* < 0.05 ## *p* < 0.01 ### *p* < 0.001, vs. agPRP.

**Table 2:** PFP thrombin generation parameters.

	**Lag Time (s)**			
	**PFP**	**PFP + Rivaroxaban 15 ng/mL**	**PFP + Rivaroxaban 50 ng/mL**	**PFP + Rivaroxaban 200 ng/mL**
1 µM PV	8.6 (5.2–14)	10.5 (6.8–15)	12 (7.9–17)	19 (9.6–32) *
4 µM PV	7.1 (4.6–11)	8.9 (5.9–14)	11 (6.8–16)	16 (9.8–26) **
48 µM PV	7.9 (4.6–11)	8.6 (5.9–13)	11 (7.3–19)	15 (10–22) **
	**ETP (nM.min)**			
	**PFP**	**PFP + Rivaroxaban 15 ng/mL**	**PFP + Rivaroxaban 50 ng/mL**	**PFP + Rivaroxaban 200 ng/mL**
1 µM PV	572 (387–1837)	426 (322–1150)	300 (184–663)	104 (67–332) *
4 µM VS	687 (457–2035)	616 (409–1527)	320 (210–1021)	163 (111–363) **
48 µM PV	907 (737–1878)	745 (596–1779)	516 (282–1176)	245 (144–483) **
	**Peak (nM)**			
	**PFP**	**PFP + Rivaroxaban 15 ng/mL**	**PFP + Rivaroxaban 50 ng/mL**	**PFP + Rivaroxaban 200 ng/mL**
1 µM PV	43 (30–170)	27 (17–70)	12 (10–34) *	4.2 (3.1–16) *
4 µM PV	60 (39–280)	45 (21–121)	16 (11–65)	7 (5.6–19) **
48 µM PV	100 (76–336)	67 (40–201)	29 (18–94) *	12 (9.3–33) ***
	**tt peak (s)**			
	**PFP**	**PFP + Rivaroxaban 15 ng/mL**	**PFP + Rivaroxaban 50 ng/mL**	**PFP + Rivaroxaban 200 ng/mL**
1 µM PV	18 (11–21)	21 (17–25)	26 (21–29)	34 (23–48) ***
4 µM PV	14 (11–20)	17 (11–20)	22 (14–25)	29 (20–38) **
48 µM PV	13 (7.9–15)	12.9 (9.9–19)	18 (12–27)	25 (19–32) ***
	**Velocity (nM/min)**			
	**PFP**	**PFP + Rivaroxaban 15 ng/mL**	**PFP + Rivaroxaban 50 ng/mL**	**PFP + Rivaroxaban 200 ng/mL**
1 µM PV	5 (3.9–27)	2.3 (1.2–6.7)	1 (0.6–2.6) *	0.3 (0.2–1.1) ***
4 µM PV	8.4 (5–64)	6.3 (2.1–22)	2.3 (0.9–11)	0.6 (0.4–1.8) **
48 µM PV	23 (16–100)	16 (4.2–50)	4.9 (2.3–19)	1.3 (0.8–4.0) ***

Thrombin generation parameters of platelet free plasma (PFP) triggered with 1 pM of TF with addition of rivaroxaban and phospholipid vesicles (PV). Results are presented as median (min-max). *n* = 6, * *p* < 0.05, ** *p* < 0.01, *** *p* < 0.001, vs. PFP.

## Supplementary Materials

The following are available online at https://www.mdpi.com/2077-0383/8/11/1990/s1, Figure S1: Plasma preparations from human citrated blood samples for CAT assay; Figure S2: IgG isotype control effect on aggregated platelet in TG.
