# Supplementary material for: Rivaroxaban Effects Illustrate the Underestimated Importance of Activated Platelets in Thrombin Generation Assessed by Calibrated Automated Thrombography
Source: J Clin Med. 2019 Nov 15;8(11):1990. doi: 10.3390/jcm8111990 (PMC6912513; doi:10.3390/jcm8111990)
Supplement: Supplementary file 1 [file jcm-08-01990-s001.pdf]

Journal of Clinical Medicine

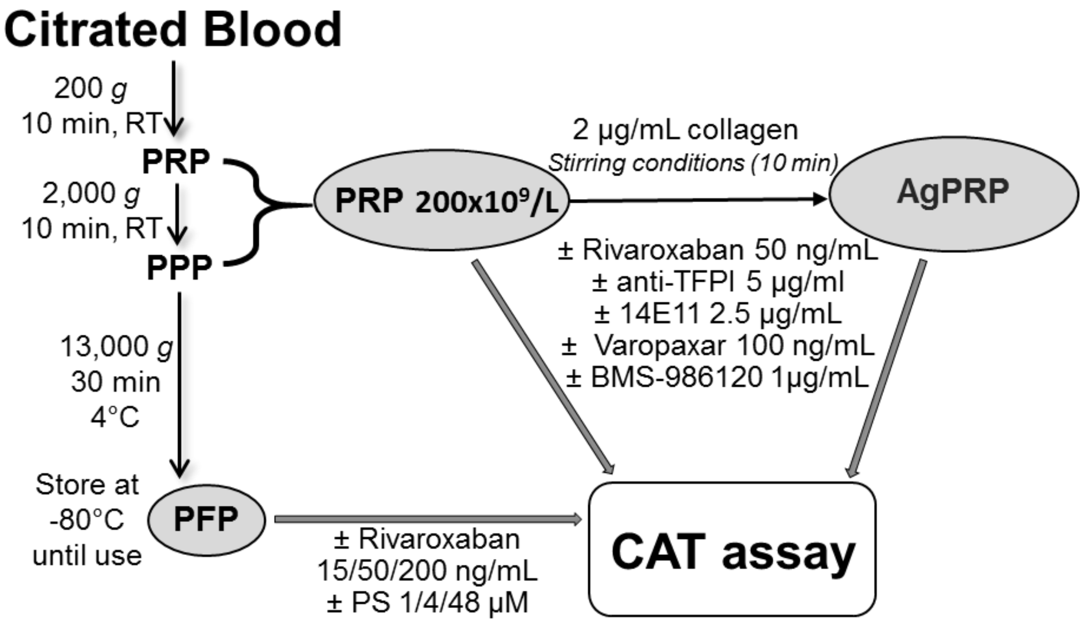

Figure S1. Plasma preparations from human citrated blood samples for CAT assay. PRP, platelet rich plasma; PPP, platelet-poor plasma; PFP, platelet free plasma; AgPRP, aggregated PRP; RT, room temperature; CAT, calibrated automated thrombography; TFPI; tissue factor pathway inhibitor.

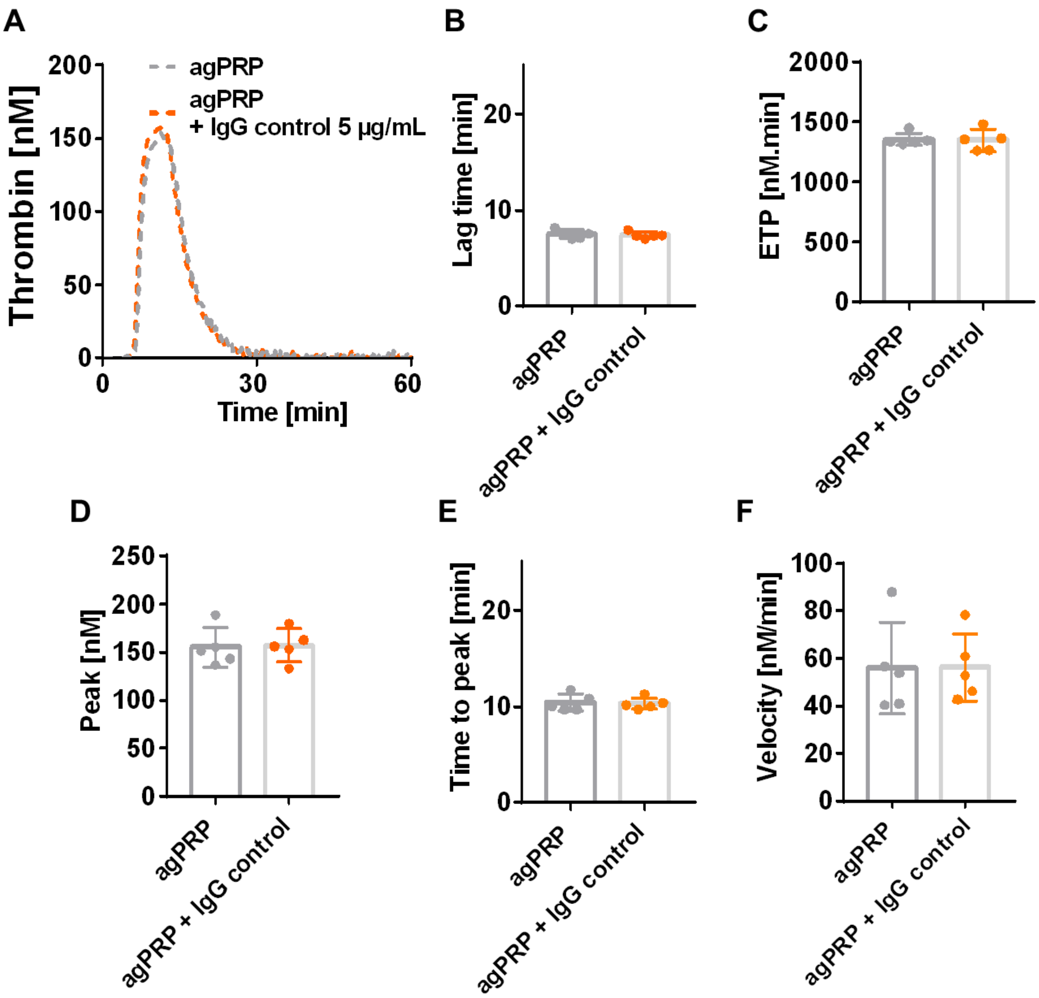

Figure S2: IgG isotype control effect on aggregated platelet in TG. Representative Thrombin generation (TG) curves in aggregated PRP (agPRP) triggered with 2 µg/mL of collagen with and without addition or 5 µg/mL IgG isotype control (**A**). TG parameters: lag time, endogenous thrombin potential (ETP); peak; time to peak and velocity (**B-F**) Results are presented as median (min-max). n = 5 per group.
